# Frequent detection of Merkel cell polyomavirus DNA in sera of HIV-1-positive patients

**DOI:** 10.1186/1743-422X-10-84

**Published:** 2013-03-13

**Authors:** Hitomi Fukumoto, Yuko Sato, Hideki Hasegawa, Harutaka Katano

**Affiliations:** 1 Department of Pathology, National Institute of Infectious Diseases, Shinjuku, Tokyo, 162-8640, Japan; 2 Japan Ground Self Defense Force, Signal School, Yokosuka, Kanagawa, 239-0828, Japan

**Keywords:** Merkel cell polyomavirus, Human polyomavirus, HPyV6, HPyV7, Real-time PCR, HIV-1

## Abstract

**Background:**

Merkel cell polyomavirus (MCPyV), human polyomavirus-6 (HPyV6), and human polyomavirus-7 (HPyV7) were identified as viruses shed from the skin. Serological analysis revealed that these viruses are common among the general population. However, there is little information about the presence of MCPyV, HPyV6, and HPyV7 in the sera and tissues of immunocompromised individuals. The aims of this study are to know if immune status affects the presence of MCPyV, HPyV6, and HPyV7 in the serum, and to reveal the presence of these viruses in diseased tissues of unknown etiology.

**Methods:**

Sera from HIV-1-positive and -negative patients were examined by real-time PCR and nested PCR detecting MCPyV, HPyV6 and HPyV7. In addition, diseased tissue samples of unknown etiology were examined.

**Results:**

Nine out of 23 serum samples (39.1%) from HIV-1-positive patients who had not received anti-retroviral therapy were positive for MCPyV, which is significantly higher than HIV-1-negative patients (6/110, 5.5%, P < 0.01, Chi-square test). MCPyV DNA was detected in tissue samples of Merkel cell carcinoma (22/30 [73%]), encephalitis (4/19 [21%]), pneumonia (3/17 [18%]), and myocarditis (8/14 [57%]). With the exception of Merkel cell carcinoma samples, MCPyV-positive tissues showed low copy numbers of MCPyV DNA by real-time PCR and no expression of the MCPyV large T antigen by immunohistochemistry. HPyV6 and HPyV7 were rarely detected in serum and tissue samples.

**Conclusions:**

These results suggest that MCPyV viremia is associated with host immunity, and that circulation of HPyV6 and HPyV7 in the serum is rare.

## Background

*Polyomavirus* is a genus of non-enveloped viruses with a circular double-stranded DNA genome of approximately 5 kb. To date, 9 polyomaviruses have been discovered in humans: BK virus [1], JC virus [2], KI virus [3], WU virus [4], Merkel cell polyomavirus (MCPyV) [5], human polyomavirus-6 and 7 (HPyV6 and HPyV7) [6], trichodysplasia spinulosa-associated polyomavirus [7], and HPyV9 [8].

MCPyV is the fifth human polyomavirus. It was identified in a patient with Merkel cell carcinoma (MCC) by using digital transcriptome subtraction [5]. MCC is a rare but aggressive neuroendocrine skin tumor, with approximately 80% of cases positive for MCPyV [9-22]. Serological tests revealed that the majority of adults are seropositive for MCPyV, with seroprevalence to the MCPyV VP1 capsid protein ranging from 46% to 88% in the general population, indicating high MCPyV prevalence among the general population [6,23-25]. A recent study revealed the serological evidence of the MCPyV primary infection in childhood [26]. MCPyV is detected not only in MCC tissues but also in several tissues including skin, oral cavity, liver, colon, lung, kidney, and saliva of patients without MCC, suggesting MCPyV is widespread in the human body [16,27]. In addition, a very high detection rate (about 90%) was reported in samples of environmental surfaces in contact with human skin by PCR, suggesting shedding of MCPyV from the skin [28]. However, detail of virus titer in the blood is unknown. It is reported that MCPyV DNA was not detected by PCR in sera from 57 immunocompetent patients [29]. Another PCR study demonstrated that 3 (15%) of 20 immunosuppressed patients were positive for MCPyV DNA in the serum [30].

In a recent study, HPyV6 and HPyV7 were isolated from skin swabs of healthy donors by using rolling circle amplification (RCA) [6]. The study suggests that infection by these viruses is common among the general population, showing a seroprevalence of 69% for HPyV6 and 35% for HPyV7 in a cohort of 95 blood donors. However, the presence of HPyV6 and HPyV7 DNA in sera has not been reported. In addition, previous studies have not found evidence for a strong association between HPyV6 or HPyV7 infection and any disease. For example, 1 study detected HPyV6 and HPyV7 DNA in only 14% and 2% of skin samples of patients with skin cancer (n = 108), respectively [31]. Other studies were unable to detect HPyV6 and HPyV7 in neuroendocrine tumors (n = 50) and MCC samples (n = 28) [32,33].

MCC occurs more frequently in HIV-1-positive patients than in immunocompetent hosts [34-37]. A study using nested PCR analysis showed that HIV-1-positive men had MCPyV DNA in the skin of forehead more frequently than HIV-1-negative healthy controls [38]. In addition, reactivation of human polyomaviruses such as KI polyomavirus and WU polyomavirus was demonstrated in immunocompromised hosts [39]. However, little information about the presence of MCPyV, HPyV6, and HPyV7 in the sera of immunocompromised individuals is available. Moreover, these viruses have not been examined in samples of patients with diseases of unknown etiology. In the present study, sera from HIV-1-positive and -negative patients were examined by real-time PCR and nested PCR to know if immune status affects the presence of MCPyV, HPyV6, and HPyV7 in the serum. In addition, diseased tissue samples of unknown etiology were examined.

## Results

### Frequent detection of MCPyV in sera of HIV-1-positive patients

**Figure 1:**
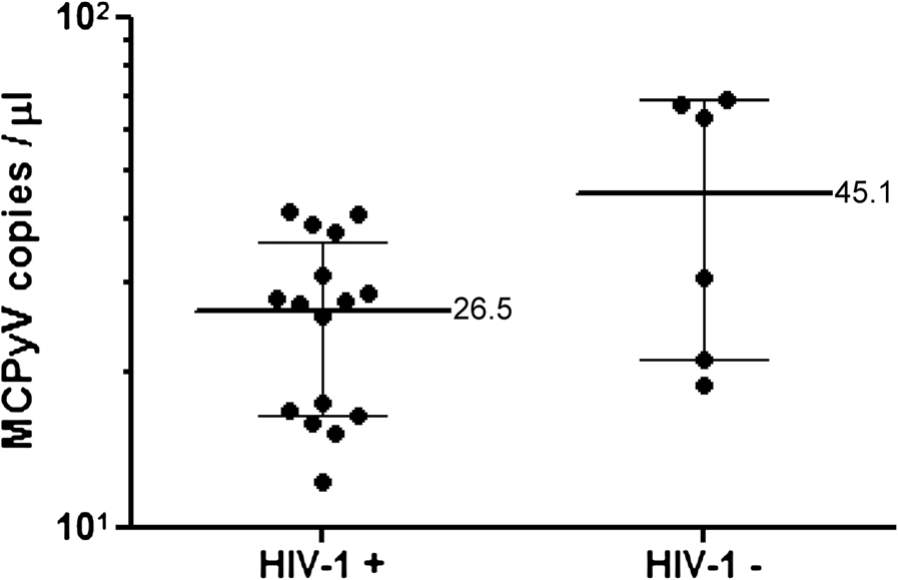
**MCPyV copy number in serum samples from HIV-1+ and HIV-1- patients.** MCPyV copy number per μL of serum is shown. Horizontal and vertical bars indicate the mean and standard deviation, respectively. The mean MCPyV copy number per μL is shown in the right of each bar.

**Figure 2:**
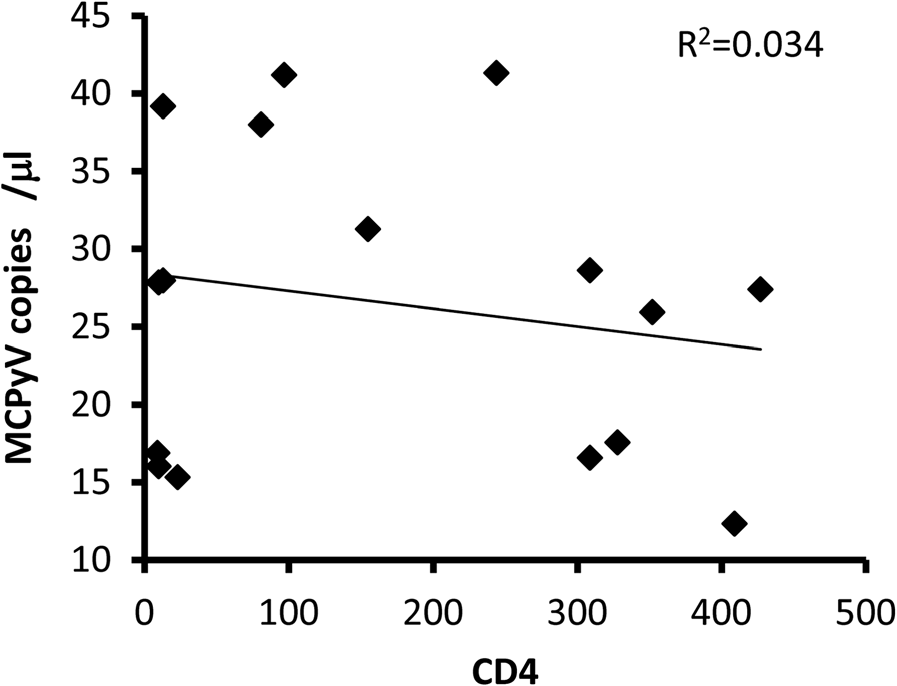
**MCPyV copies and CD4 count.** Each dot plot indicates MCPyV copy number in the serum and CD4 count per μL of blood in each HIV-1+ patient. A regression line is shown with regression coefficient (0.034).

MCPyV was detected in 9 of 23 HIV-1-positive sera (39.1%) and 6 of 110 HIV-1-negative sera (5.5%) by real-time PCR (Table 1). The positivity of MCPyV among HIV-positive patients was significantly higher than that among HIV-1-negative patients (P < 0.01, Chi-square test). In MCPyV-positive sera, there was no significant difference in MCPyV copy number between HIV-1-positive (mean = 26.5 copies per μL) and HIV-1-negative patients (mean = 45.1 copies per μL, P = 0.09, Mann-Whitney *U*-test) (Figure 1). Six MCPyV-positive sera in HIV-1-negative patients were two from congenital immunodeficiency patients, one from a sudden death patient with unknown etiology, and three from myocarditis patients. Dot blots on CD4 and MCPyV copy revealed that CD4 counts in the HIV-1-positive patients were not correlated with MCPyV copy numbers in the serum (R^2^ = 0.034, Figure 2). HPyV6 ST DNA was also detected by real-time PCR in 0 and 2 cases of HIV-1-positive and HIV-1-negative sera, respectively. The two HPyV6 positive HIV-1-negative sera which were taken from autopsy cases of elder patients with respiratory failure were negative for MCPyV. HPyV7 was not detected in any serum sample by real-time PCR.

**Table 1:** MCPyV, HPyV6, and HPyV7 detection in serum samples by real-time PCR

**Patient**	**n**	**MCPyV**	**HPyV6**	**HPyV7**
**HIV+**	23	9 (39.1%)	0 (0%)	0 (0%)
**HIV-**	110	6 (5.5%)	2 (1.8%)	0 (0%)

### MCPyV, HPyV6, and HPyV7 detection in tissue samples

**Figure 3:**
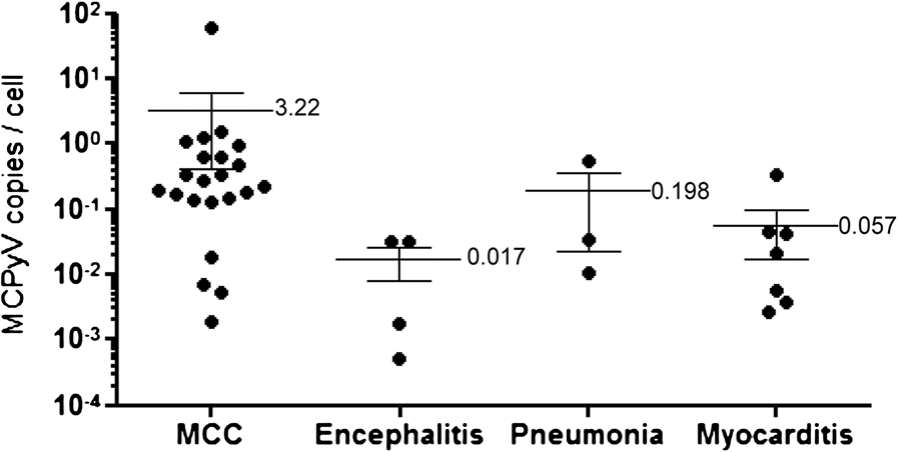
**MCPyV copies per cell in tissue samples.** The mean MCPyV copy number per cell is shown in the right of each bar. Error bars indicate standard error.

**Figure 4:**
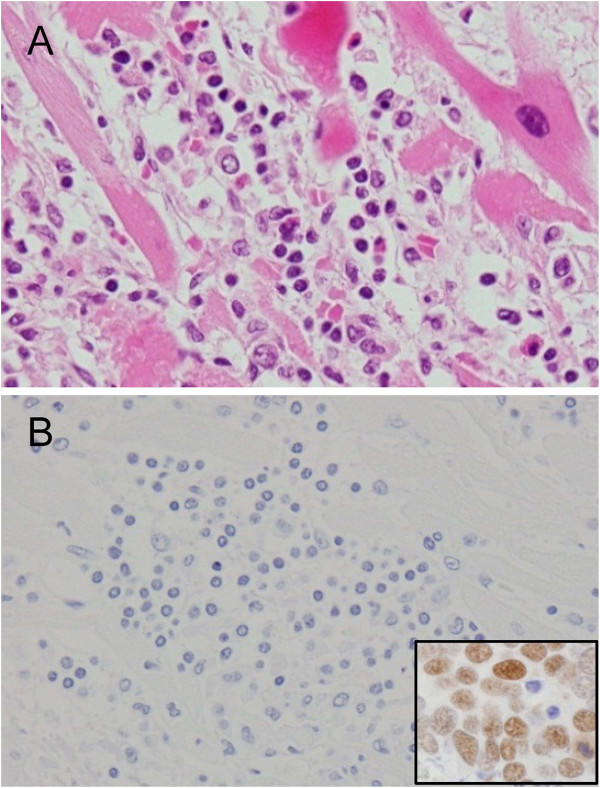
**Immunohistochemistry for MCPyV in tissue samples.** (**A**) Hematoxylin and eosin staining of a sample of a patient with myocarditis positive for MCPyV by real-time PCR. Remarkable inflammatory cell infiltration is observed in the muscle of the heart. (**B**) MCPyV large T antigen cannot be detected by immunohistochemistry in the inflammatory cells and myocardial cells. Inset indicates a positive control of Merkel cell carcinoma which had 0.7 copy per cells of MCPyV by real-time PCR.

MCPyV was detected in 22/30 (73%) tissue samples of MCC, 4/19 (21%) of encephalitis, 3/17 (18%) of pneumonia, 8/14 (57%) of myocarditis, and 0/10 (0%) of hepatitis by real-time PCR (Table 2). There was no specific histological difference between MCPyV-positive and negative cases in each group. Eleven MCPyV-positive samples of other group in Table 2 were composed of 6 AIDS-associated Kaposi’s sarcoma, 4 AIDS-associated lymphoma, and one AIDS-associated progressive multifocal leukoencephalopathy cases. Among MCPyV-positive tissues, the mean MCPyV copy number was significantly higher in the MCC tissues (3.219 copy per cell) than in the other tissues (0.075 copy per cell, P = 0.0014, Mann-Whitney *U*-test, Figure 3). The MCPyV large T antigen was detectable by immunohistochemistry only in the MCC samples (Figure 4). One MCPyV-positive MCC tissue was also positive for HPyV6 ST DNA, but HPyV6 and HPyV7 were not detected in any MCPyV-negative MCC tissues, suggesting no association between MCPyV-negative MCC and HPyV6 or HPyV7 infection. One MCC sample and a tissue sample from a Kaposi’s sarcoma patient were positive for HPyV6 ST DNA by real-time PCR (Table 2). HPyV7 was not detected in any tissue sample.

**Table 2:** MCPyV, HPyV6, and HPyV7 detection in tissue samples by real-time PCR

**Disease**	**Organ**	**n**	**MCPyV**	**HPyV6 (ST)**	**HPyV7 (ST, VP1)**
**MCC**	skin	30	22 (73.3%)	1 (3.3%)	0 (0%)
**Encephalitis**	brain	19	4 (21.1%)	0 (0%)	0 (0%)
**Pneumonia**	lung	17	3 (17.6%)	0 (0%)	0 (0%)
**Myocarditis**	heart	14	8 (57.1%)	0 (0%)	0 (0%)
**Hepatitis**	liver	10	0 (0%)	0 (0%)	0 (0%)
**Other**	various	60	11 (18.3%)	1 *(1.6%)	0 (0%)

* One HPyV6-positive sample in other group is a Kaposi’s sarcoma tissue.

### Detection of HPyV6 DNA fragments in nested PCR

**Figure 5:**
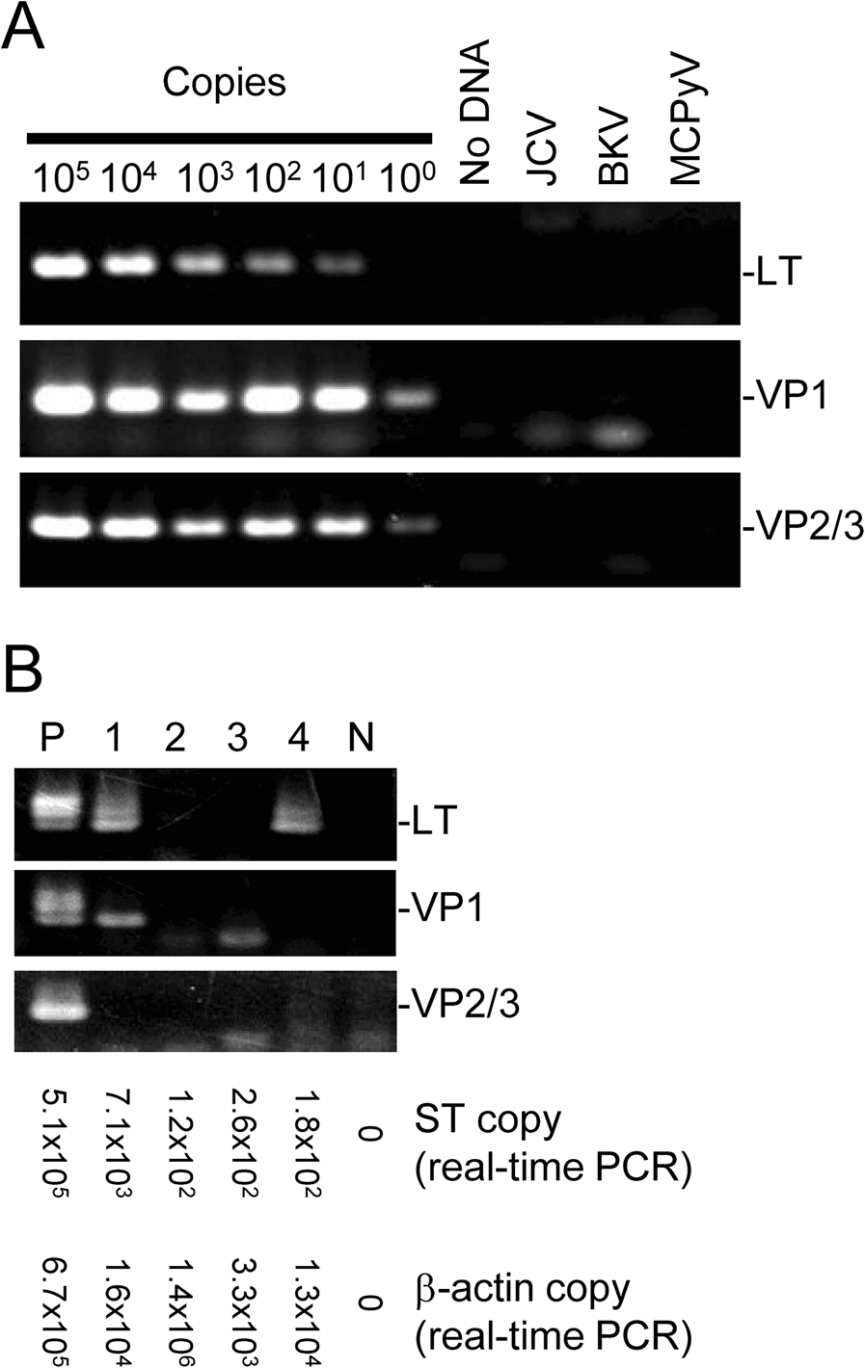
**Nested PCR and real-time PCR detecting HPyV6-encoded genes.** (**A**) Sensitivity and specificity of the nested PCR detecting three HPyV6 gene fragments were confirmed. Sequence-validated PCR products of HPyV6 were amplified by the nested PCR. In addition, JCV and BKV-positive controls were examined. (**B**) Three HPyV6 gene fragments were detected in tissue and serum samples positive for HPyV6 by real-time PCR. A case of MCC (lane 1), Kaposi’s sarcoma (lane 2), and two sera from HIV-1-negative patients (lane 3 and 4) were examined. Average copy numbers of three experiments for HPyV6 ST and human β-actin genes per 100 ng of DNA (tissue samples) or DNA extracted from 1 μL of serum were shown in the bottom. Specific PCR fragments, including large T (LT), VP1, and VP2/3, were not amplified constantly in lane 1-4. Similar results were obtained in a repeated experiment. P: positive control of HPyV6, a DNA sample extracted from a skin biopsy which was confirmed to be HPyV6-positive, N: no DNA.

We carried out nested PCR analysis on two serum and two tissue samples which were positive for HPyV6 by real-time PCR detecting HPyV6 ST DNA. The nested PCR were able to amplify their targets from ten genome copies constantly, and did not cross-react with JCV, BKV, and MCPyV genomes (Figure 5A). Nested PCR analysis revealed that HPyV6 VP2/3 DNA were failed to detect in all these cases, whereas a positive control of HPyV6, a DNA sample extracted from a skin biopsy which was confirmed to be HPyV6-positive, was positive for VP2/3 (Figure 5B). HPyV6 LT DNA was negative in the one serum and one tissue samples, and VP1 was positive only in one MCPyV-positive MCC case by nested PCR.

## Discussion

In the present study, we detected MCPyV DNA more frequently in the sera of HIV-1-positive patients than in the sera of HIV-1-negative patients. Although detailed characteristics of the HIV-1-positive patients were not available, we confirmed that these patients were not receiving antiretroviral therapy and more than half of them developed AIDS at the time of blood collection, suggesting that the patients were immunocompromised. Although the correlation between CD4 counts and MCPyV copies was not shown in the present study (Figure 2) and the detailed immune status was not determined in the HIV-1-negative patients, the significantly different positivity of MCPyV DNA in the serum between HIV-1-positive and negative patients suggests that an individual’s immune status is associated with the production of MCPyV which will be a crucial factor in elucidating MCC pathogenesis. It has been reported that profound immune suppression is one of the important risk factors for MCC development and that patients with AIDS have a 13-fold increased risk for MCC compared with the general population [35]. Thus, a high detection rate of MCPyV in the serum may be associated with the clinical incidence of MCC.

MCPyV, HPyV6, and HPyV7 were identified as viruses shed from the skin [6]. Seroprevalence data from a cohort of U.S. patients indicates that these 3 viruses are common among the general population [6,40]. In contrast, our PCR data showed the presence of viral DNA in less than 10% of serum samples from HIV-1-negative patients. This finding is consistent with the results by previous reports describing frequent detection of MCPyV in the skin samples [6], but rare detection of MCPyV DNA in blood samples [29]. Considering these reports and our results, it is suggested that viremia is rare among infected individuals who are not immunocompromised. Although the seroprevalence of the viruses in Japan is unknown, low detection rates of the viruses in serum suggest a low titer of circulating virus or low amounts of viral DNA in the blood of infected individuals. A low detection rate of viral DNA in MCPyV-seropositive individuals would suggest that the viruses are produced in the skin and released from the epithelium, but do not circulate in the blood. Because HPyV6 and HPyV7 were rarely detected even in HIV-1-positive serum samples, seroprevalence data on these viruses will be required to interpret the results. In addition, HPyV6 was detected in 4 samples including tissue samples in the present study, but only 1 MCC tissue was positive for both VP1 and ST genes. HPyV6 LT DNA was negative in the one serum and one tissue samples by the nested PCR among the 4 samples positive for HPyV6 ST gene by the real-time PCR. Such different reactivities among polyomavirus-encoded genes in each case were observed in the previous reports of MCPyV in MCC [20,22], suggesting sequence mutation in the target genes or the presence of partial genomes of HPyV6 in the serum.

MCPyV was detected at a high rate in samples of patients with myocarditis (57%); additionally, 10–20% of pneumonia and encephalitis samples were positive. Our results in the present study showed low copy numbers of MCPyV in tissues other than MCC. These findings are constant to previous findings that no or low amounts of MCPyV was detected in normal tissues or neoplastic lesions of organs other than the skin [22]. The previous study demonstrated that MCC cases infected with MCPyV at more than 0.05 copy per cell were positive in immunohistochemistry for MCPyV large T antigen [41]. Although more than 0.05 copy per cell of MCPyV were detected in one pneumonia and one myocarditis cases, immunohistochemistry showed no expression of the MCPyV large T antigen in tissues other than MCC tissues, indicating no direct association of MCPyV infection with the pathogenesis of these diseases. However, since MCPyV was detected in inflammatory conditions such as myocarditis and pneumonia by real-time PCR in the present study, MCPyV production may be induced by inflammations or inflammatory cytokines. This observation is compatible with a recent finding that inflammatory monocyte is a reservoir for MCPyV in vivo [42]. Another possibility is that the virus has an inherent affinity for heart or lung cells. Further investigation of the virus receptor will be required to determine its affinity.

## Conclusions

MCPyV DNA was detected more frequently in the sera of HIV-1-positive patients than in the sera of HIV-1-negative patients. HPyV6 was detected in less than 2% of serum and tissue samples, whereas HPyV7 was not detected. These results suggest that MCPyV replication is associated with host immunity, and that circulation of HPyV6 and HPyV7 in the serum is rare.

## Methods

### Samples

This study was approved by the institutional review board at the National Institute of Infectious Diseases (Approval No. 273). We used 23 HIV-1-positive and 111 HIV-1-negative sera stored at the National Institute of Infectious Disease. All the HIV-1-positive patients had not received anti-retrovirus therapy at the time of blood collection. Their CD4 counts at the time of blood collection were recorded for analysis. The HIV-1-negative sera were obtained from patients with various diseases including influenza virus infection, myocarditis, encephalitis, hepatitis, malignancies, etc. No MCC patient was included. In addition, formalin-fixed paraffin-embedded (FFPE) or frozen tissue samples from 150 patients with various diseases such as encephalitis, pneumonia, myocarditis, and hepatitis were collected. DNA samples extracted from JCV-positive progressive multifocal leukoencephalopathy and BKV-associated nephropathy were used as JCV and BKV-positive controls, respectively.

### DNA extraction

DNA was extracted from 50 μL of serum by using the DNeasy Blood & Tissue Kit (Qiagen GmbH, Hilden, Germany) according to the manufacturer’s protocol. DNA from sera was eluted in a final volume of 50 μL of elution buffer. For tissue samples, DNA was extracted from three pieces of 10 μm-thick FFPE sections and 10 mg of frozen tissue samples with the Qiaamp FFPE DNA extraction kit and the DNeasy Tissue Kit (Qiagen), respectively. DNA from FFPE and frozen tissues were eluted in a final volume of 30 μL and 100 μL of elution buffer, respectively.

### Real-time PCR

Real-time PCR was performed using a standard TaqMan® PCR kit protocol (Applied Biosystems, Foster City, CA) on a MX3005P (Stratagene, La Jolla, CA). DNA samples were analyzed for the presence of MCPyV [22], HPyV6 VP1, HPyV6 small T (ST), HPyV7 VP1, and HPyV7 ST genes. The amount of human genomic DNA (as measured by the β-actin gene) in the DNA extracted from each specimen was also determined. Primers and probes for HPyV6 and HPyV7 were designed for the VP1 and ST regions by using Primer Express software (Applied Biosystems) based on the reference sequences of HPyV6 (GenBank accession no. HM011558) and HPyV7 (HM011569). HPyV6-VP1 forward (5^′^-CCCTGGCTGTTGTTAATTTGC-3^′^) and reverse (5^′^-CTGAAGGCTTCCCAAACCAA-3^′^) primers were used with the TaqMan probe 5^′^-(FAM) TGAAATTCCTGAGGCCCTGTGTGATGAT (TAMRA)-3^′^. HPyV6-ST forward (5^′^-AAGCACCAGGTGGGTGATGA-3^′^) and reverse (5^′^-CAACGCCTGAATGTTTTAAAGGA-3^′^) primers were used with the TaqMan probe 5^′^-(FAM) TTGGTCCCTCAGGGTGGCATTCAA (BHQ1)-3^′^. HPyV7-VP1 forward (5^′^-AGAAGGTCCAGGCAATAGTGATG-3^′^) and reverse (5^′^-CTGGGAAATTTGCAGCATTTACT-3^′^) primers were used with the TaqMan probe 5^′^-(HEX) AGCTAGCCTGCAAGCCCTCAGAAAGC (BHQ1)-3^′^. HPyV7-ST forward (5^′^-CCAGCATTTGCCCCATAAAA-3^′^) and reverse (5^′^- AAAGCATAAGAAGAAGGCCAAAGA-3^′^) primers were used with the TaqMan probe 5^′^-(HEX) AGGCCCCCGGTGGTCTTTAG (BHQ1)-3^′^. Primers and probes for MCPyV and β-actin were described previously [22]. PCR amplification was performed in a 20 μL reaction volume by using QuantiTect Multiplex PCR Master Mix (Qiagen), with 0.4 μM of each primer, 0.2 μM of TaqMan probe, and 1 μL of isolated DNA. PCR was carried out at 50°C for 2 min, 95°C for 15 min, and 40 cycles of 94°C for 1 min and 60°C for 1 min. Quantitative results were obtained by generating standard curves for sequence-validated PCR products or plasmids containing HPyV6-VP1, HPyV6-ST, HPyV7-VP1, HPyV7-ST, MCPyV-LT, or the cellular target (β-actin gene). Virus copy number per cell was calculated as previously described, by dividing the virus copy number by half of the β-actin copy number, because each cell contains 2 alleles of β-actin [43]. These real-time PCR amplified at least 10 copies of target gene constantly, and did not cross-react with JCV and BKV in JCV or BKV-positive control samples (data not shown). In addition, HPyV6 and HPyV7 real-time PCR did not amplify any fragment from a plasmid containing a full genome of MCPyV (data not shown).

### Nested PCR

Nested PCR was performed to detect HPyV6 LT, VP1, and VP2/3 gene. Sequences of outer and inner primers are listed in Table 3. The first round of amplification was performed with 100 ng of extracted DNA and high fidelity Taq DNA polymerase (Roche Diagnostics, Boehringer Mannheim, Germany) in a final volume of 25 μL. After an initial DNA denaturation for 2 min at 94°C, samples were amplified by 35 cycles of 94°C for 30 sec, 55°C for 30 sec and 72°C for 30 sec, followed by a final elongation phase of 7 min at 72°C. The second round was performed with 1 μL of first round PCR product in a final volume of 25 μL under the following parameters: 94°C for 30 sec, 55°C for 30 sec, 72°C for 30 sec for 25 cycles, followed by a final elongation phase of 7 min at 72°C. Five μL of amplification products were loaded onto agarose gels, electrophoresed, stained with bromide and visualized under UV light.

**Table 3:** Primer list for HPyV6 nested PCR

**Gene**	**Out/In**	**F/R**	**Primer name**	**5**^**′**^**-3**^**′**^	**Product Size**
HPyV6 LT	Outer	Forward	HPyV6-LTN4088F1	ggagcaggattgggttttct	203 bp
		Reverse	HPyV6-LTN4290R1	aggccacctccacaatatgg	
	Innner	Forward	HPyV6-LTN4109F2	ttcttaggaggagtgcaaga	149 bp
		Reverse	HPyV6-LTN4257R2	gaacaatggtgggctgattt	
HPyV6 VP1	Outer	Forward	HPyV6-VP1-1347 F1	ggaggagtggaggttatgga	166 bp
		Reverse	HPyV6-VP1-1512R1	acagagatgaaccagcatcc	
	Innner	Forward	HPyV6-VP1-1369 F2	cagtgccactttctgaagac	122 bp
		Reverse	HPyV6-VP1-1490R2	agtgtcggtaaaggtgtagg	
HPyV6 VP2/3	Outer	Forward	HPyV6-VP23-741 F1	cacttcaactgtggttgcca	187 bp
		Reverse	HPyV6-VP23-927R1	tccctagaagctgttctctg	
	Innner	Forward	HPyV6-VP23-763 F2	agtttggtcttggggaggcg	138 bp
		Reverse	HPyV6-VP23-900R2	tccctgcctgctctatagta	

### Immunohistochemistry

Immunohistochemistry was performed on FFPE samples with the rabbit anti-MCPyV-LT polyclonal antibody as the primary antibody, as described previously [41].

### Statistical analysis

Data were analyzed using a Chi-square test or Mann-Whitney *U*-test on SPSS software (IBM, Armonk, NY).

## Competing interests

The authors declare that they have no competing interests.

## Authors’ contributions

HK designed the study and performed statistical analysis. HF and HK performed PCR. YS, HK and HH performed pathological analysis. HK and FH drafted the manuscript. All authors read and reviewed the final manuscript.
